# Recent Advances in Smart Wound Healing Patches: From Passive Dressings to Intelligent Theranostic Systems

**DOI:** 10.7759/cureus.114607

**Published:** 2026-08-16

**Authors:** Rahul A Sachdeo, Akshay R Yadav, Chitra Khanwelkar, Amol Shete

**Affiliations:** 1 Department of Pharmacology, Krishna Institute of Medical Sciences, Krishna Vishwa Vidyapeeth (Deemed to be University), Karad, IND; 2 Department of Pharmaceutical Chemistry, Krishna Charitable Trust's Krishna College of Pharmacy, Karad, IND; 3 Department of Pharmaceutics, Krishna Institute of Pharmacy, Krishna Vishwa Vidyapeeth (Deemed to be University), Karad, IND

**Keywords:** controlled drug delivery, regenerative medicine, smart wound care, theranostic systems, wearable biosensors, wound healing patches

## Abstract

Wound healing remains a complex and multifaceted physiological process, especially in the treatment of chronic wounds such as diabetic ulcers, burns, and pressure injuries. Conventional wound dressings work primarily as passive barriers, failing to provide dynamic interaction with the wound milieu, resulting in delayed healing and an increased risk of infection. Recent breakthroughs in biomaterials science, nanotechnology, and bioengineering have resulted in the creation of next-generation wound healing patches with multifunctional properties. These include hydrogel-based systems, bioactive and nanocomposite dressings, microneedle patches, and smart wearable platforms with real-time monitoring and controlled medication delivery. Smart and stimuli-responsive wound patches have emerged as viable solutions, allowing for adaptive therapeutic responses to wound-specific factors such as pH, temperature, and reactive oxygen species. Furthermore, the combination of biosensors with wearable electronics has enabled continuous, non-invasive monitoring of wound states, allowing for individualized and data-driven treatment plans. Theranostic wound patches, which combine diagnostic and therapeutic capabilities on a single platform, represent a major paradigm shift in wound care. Despite these advances, obstacles such as high costs, regulatory constraints, insufficient clinical validation, and long-term safety concerns impede widespread clinical implementation. Future advancements are projected to center on the integration of artificial intelligence, self-powered systems, 3D bioprinting, and regenerative therapies to enable entirely autonomous and individualized wound management. This study gives a thorough account of recent breakthroughs in wound healing patches, emphasizing material innovations, technological advancements, clinical uses, and future prospects in this rapidly growing subject.

## Introduction and background

Hemostasis, inflammation, proliferation, and tissue remodeling are components of the highly dynamic, multistage biological process of wound healing, which is regulated by complex cellular and molecular interactions [1,2]. Managing acute and chronic wounds, such as diabetic ulcers, pressure sores, and burn injuries, continues to be a significant global healthcare concern despite advancements in clinical treatment. Millions of people worldwide suffer from chronic wounds, which are often linked to microbial infection, inadequate angiogenesis, persistent inflammation, and delayed tissue regeneration. These factors raise morbidity and medical costs. Traditional wound dressings, such as gauze and cotton-based materials, protect the wound site and absorb exudates by acting as passive physical barriers. However, these traditional methods are incapable of dynamically modulating the wound microenvironment, delivering therapeutic drugs, or providing real-time feedback on wound condition. Furthermore, their failure to maintain adequate moisture balance, to stimulate cellular activity, or to prevent infection reduces their therapeutic usefulness in complex wound situations [3-5]. Even early-generation hydrogel dressings, while biocompatible and moisture-retentive, are generally passive and lack dynamic response to physiological changes in the wound environment. Recent advances in nanotechnology, bioengineering, and biomaterials research have sparked a paradigm shift toward the development of next-generation wound healing patches with versatile and multipurpose properties. Because they may mimic the extracellular matrix (ECM), encourage cell proliferation, and provide controlled drug delivery, hydrogel-based systems, nanofiber scaffolds, microneedle patches, and bioactive polymeric matrices have all been the subject of much investigation. The remarkable expansion of hydrogel research, which has more than quadrupled since 2020, is indicative of a growing interest in creating multifunctional, stimuli-responsive wound dressings that can simultaneously treat inflammation, infection, and tissue regeneration [6]. An important development in this area is the creation of smart and theranostic wound patches, which combine therapeutic and diagnostic sensing capabilities. In order to monitor vital wound indicators like pH, temperature, glucose levels, and reactive oxygen species (ROS) and then initiate regulated medication release or therapeutic reactions, these systems are built with stimuli-responsive biomaterials [7]. The incorporation of wearable electronics and biosensors into wound patches has allowed for real-time, non-invasive monitoring of wound progression, permitting individualized and timely therapies. Furthermore, new technologies like 3D bioprinting and AI-assisted wound assessment are paving the road for personalized, patient-specific wound care solutions. Collectively, these improvements represent a shift from passive wound coverage to intelligent, multifunctional platforms capable of actively managing the wound microenvironment and expediting recovery. This review seeks to critically examine recent breakthroughs in wound healing patches, with a focus on material innovations, design tactics, and new theranostic systems [8,9].

## Review

Mechanism of wound healing

Hemostasis, inflammation, proliferation, and remodeling are all stages that overlap in the highly coordinated and dynamic biological process of wound healing. Complex interactions between various cell types, cytokines, growth hormones, and ECM components control this process. Healing can be hampered by any interference with these carefully regulated processes, particularly in chronic wounds [10].

Hemostasis Phase

In order to stop excessive blood loss, the hemostasis phase begins right after tissue damage. At the site of damage, platelet adhesion and aggregation happen after rapid vasoconstriction. A fibrin-rich clot that serves as a temporary ECM scaffold is created when activated platelets release clotting proteins. Key growth factors, including PDGF and transforming growth factor-beta (TGF-β), which are crucial for attracting immune cells and starting later stages of healing, are secreted by platelets in addition to coagulation. This temporary matrix also facilitates cell migration and provides structural stability to the wound site [11,12].

Inflammatory Phase

After an injury, the inflammatory phase usually lasts for a few days and starts within hours. The infiltration of immune cells, mostly neutrophils and macrophages, is what defines it. As the initial line of defense, neutrophils eliminate pathogens and cellular debris by phagocytosing them and producing ROS. This phase is thereafter dominated by macrophages, which are crucial for regulation [13]. They release cytokines and growth factors, including fibroblast growth factor (FGF) and vascular endothelial growth factor (VEGF), as they go from a pro-inflammatory (M1) to an anti-inflammatory (M2) phenotype. These mediators support tissue healing, angiogenesis, and inflammation resolution. One of the main characteristics of chronic wounds is persistent or dysregulated inflammation [14].

Proliferation Phase

Granulation tissue development, re-epithelialization, and angiogenesis are all part of the proliferation phase. In order to create a new structural framework, fibroblasts move into the wound site and produce ECM components like collagen (mostly type III), fibronectin, and proteoglycans. Growth factors like VEGF promote angiogenesis, which results in the formation of new capillaries that replenish the supply of nutrients and oxygen. In order to restore the epithelial barrier, keratinocytes multiply and move across the wound area at the same time. This stage is essential for both functional tissue regeneration and wound closure [15,16].

Remodeling (Maturation) Phase

The ECM is strengthened and reorganized throughout the remodeling phase, which can last anywhere from a few weeks to many months. The healed tissue's tensile strength increases when type I collagen increasingly replaces type III collagen. Collagen remodeling and breakdown are controlled by matrix metalloproteinases (MMPs) and their tissue inhibitors. Myofibroblasts, which aid in wound contraction, develop from fibroblasts. However, fibrosis or the development of hypertrophic scars can result from excessive collagen deposition or dysregulated remodeling [17-19].

Advanced wound patches are designed to support and enhance these healing phases by maintaining a moist wound environment, controlling exudate, preventing microbial contamination, and facilitating oxygen exchange. Furthermore, modern wound patches can serve as delivery platforms for bioactive agents such as growth factors, antimicrobials, stem cells, and therapeutic drugs, thereby promoting cellular proliferation, angiogenesis, tissue regeneration, and efficient wound remodeling. Thus, advanced wound patches play a crucial role in optimizing the wound microenvironment and accelerating the overall healing process.

The wound healing cascade consists of multiple overlapping stages, each characterized by distinct cellular activities and physiological events that collectively contribute to tissue restoration, as summarized in Table 1.

**Table 1:** Mechanism and stages of wound healing process

Stage of wound healing	Key events	Major cells involved	Role in healing process
Hemostasis	Blood clot formation and vasoconstriction occur immediately after injury	Platelets, endothelial cells	Prevents blood loss and forms temporary protective barrier [20]
Inflammation	Release of cytokines and immune response activation	Neutrophils, macrophages, lymphocytes	Removes debris, pathogens, and damaged tissue [21,22]
Proliferation	Formation of granulation tissue, angiogenesis, and collagen deposition	Fibroblasts, keratinocytes, endothelial cells	Promotes tissue regeneration and wound closure [23]
Epithelialization	Migration and proliferation of epithelial cells over wound surface	Keratinocytes	Restores skin barrier and surface integrity [24]
Angiogenesis	Development of new blood vessels in wound area	Endothelial cells	Supplies oxygen and nutrients to regenerating tissue [25]
Collagen remodeling	Replacement of immature collagen with mature collagen fibers	Fibroblasts	Enhances tensile strength and tissue organization [26,27]
Maturation/remodeling	Scar formation and tissue strengthening over time	Fibroblasts, myofibroblasts	Restores functional integrity of healed tissue [28,29]

Impaired Wound Healing in Chronic Conditions

Diabetic foot ulcers and other chronic wounds show slow recovery. Persistent inflammation, decreased angiogenesis, decreased growth factor activity, and an elevated microbial burden-often linked to biofilm formation-are the hallmarks of these wounds. These disease disorders cause delayed or non-healing wounds by interfering with ECM remodeling and cellular signaling. Advanced wound healing patches are specifically designed to address these challenges by modulating the wound microenvironment, promoting tissue regeneration, and enabling targeted therapeutic interventions [30].

Types of advanced wound healing patches

Multifunctional wound healing patches that actively control the wound microenvironment have been made possible by recent developments in biomaterials and bioengineering. These systems are intended to maintain moisture balance, provide regulated drug delivery, prevent infection, and stimulate tissue regeneration [31]. Based on their composition and functional mechanisms, advanced wound healing patches can be broadly divided into the following groups.

Hydrogel-Based Patches

One of the most cutting-edge and thoroughly researched biomaterials in modern wound treatment is hydrogel-based wound healing patches. Large volumes of water can be absorbed and retained by hydrogels, which are three-dimensional polymeric networks composed of hydrophilic materials without compromising their structural integrity [32]. Because of their high water content and soft consistency, hydrogels closely resemble the ECM found in human tissues, creating an environment that is ideal for wound healing. They are perfect for treating burns, diabetic ulcers, acute and chronic wounds, and post-operative injuries because of their unique structural and physicochemical characteristics. The ability of hydrogel-based patches to provide a moist wound environment, which is necessary for efficient tissue healing and regeneration, is one of their most important advantages. Moist wounds promote epithelialization, minimize scar formation, and facilitate cellular migration and proliferation. Hydrogels also offer high oxygen permeability while protecting the wound from microbial invasion and external pollutants. Furthermore, their cooling impact helps to reduce pain and inflammation in burn wounds and highly sensitive lesions. Both synthetic and natural polymers can be used to make hydrogels. Because of their excellent biocompatibility, biodegradability, and intrinsic biological activity, natural polymers like chitosan, alginate, gelatin, collagen, hyaluronic acid, and cellulose derivatives are employed extensively [33]. Chitosan has antibacterial and hemostatic characteristics, whereas alginate is better at absorbing exudate and producing gels. Hyaluronic acid stimulates angiogenesis and collagen deposition, whereas gelatin encourages cell adhesion and tissue regeneration. However, natural polymers frequently exhibit low mechanical strength and quick breakdown. To address these constraints, hydrogel formulations include synthetic polymers such as polyvinyl alcohol (PVA), polyethylene glycol (PEG), polyacrylamide, and polyurethane. These polymers offer increased mechanical stability, elasticity, and regulated breakdown rates. Hybrid hydrogels made from natural and synthetic polymers have received a lot of attention because they combine biological functionality with better structural performance. Recent advances in hydrogel engineering have resulted in the creation of smart, multifunctional hydrogel systems. Double-network hydrogels have greater mechanical resilience and toughness due to the interpenetration of two polymeric networks. Conductive hydrogels containing graphene, polypyrrole, or metallic nanoparticles can send electrical signals that promote cell growth and tissue repair. Injectable hydrogels are administered directly into the wound bed through minimally invasive injection, allowing them to conform to irregular wound geometries and fill wound cavities effectively. Stimulus-responsive hydrogels are another significant advancement in wound healing technology. These intelligent systems respond to environmental cues like pH, temperature, glucose content, enzyme activity, and ROS. For example, the acidic pH of infected wounds might cause antimicrobial compounds to be released from the hydrogel matrix. Similarly, temperature-sensitive hydrogels can control drug release based on inflammatory conditions. These adaptive drug delivery methods increase therapeutic accuracy while reducing systemic side effects [34,35]. Hydrogels are also excellent transporters for bioactive substances such as antibiotics, antioxidants, anti-inflammatory medicines, stem cells, growth factors, peptides, and nanoparticles. The encapsulation of silver nanoparticles or zinc oxide nanoparticles improves antibacterial activity against multidrug-resistant microorganisms. Growth factor-loaded hydrogels stimulate angiogenesis, fibroblast proliferation, and collagen production, speeding tissue regeneration. Despite their various benefits, hydrogel-based patches have several disadvantages, such as low mechanical endurance in highly dynamic body locations, susceptibility to dehydration, and difficulty in large-scale manufacture. Nonetheless, ongoing research in biomaterials science, nanotechnology, and tissue engineering is dramatically enhancing hydrogel performance. As a result, hydrogel-based wound dressings are predicted to play an important role in future individualized and regenerative wound care treatments [36].

Smart/Stimuli-Responsive Patches

**Figure 1:**
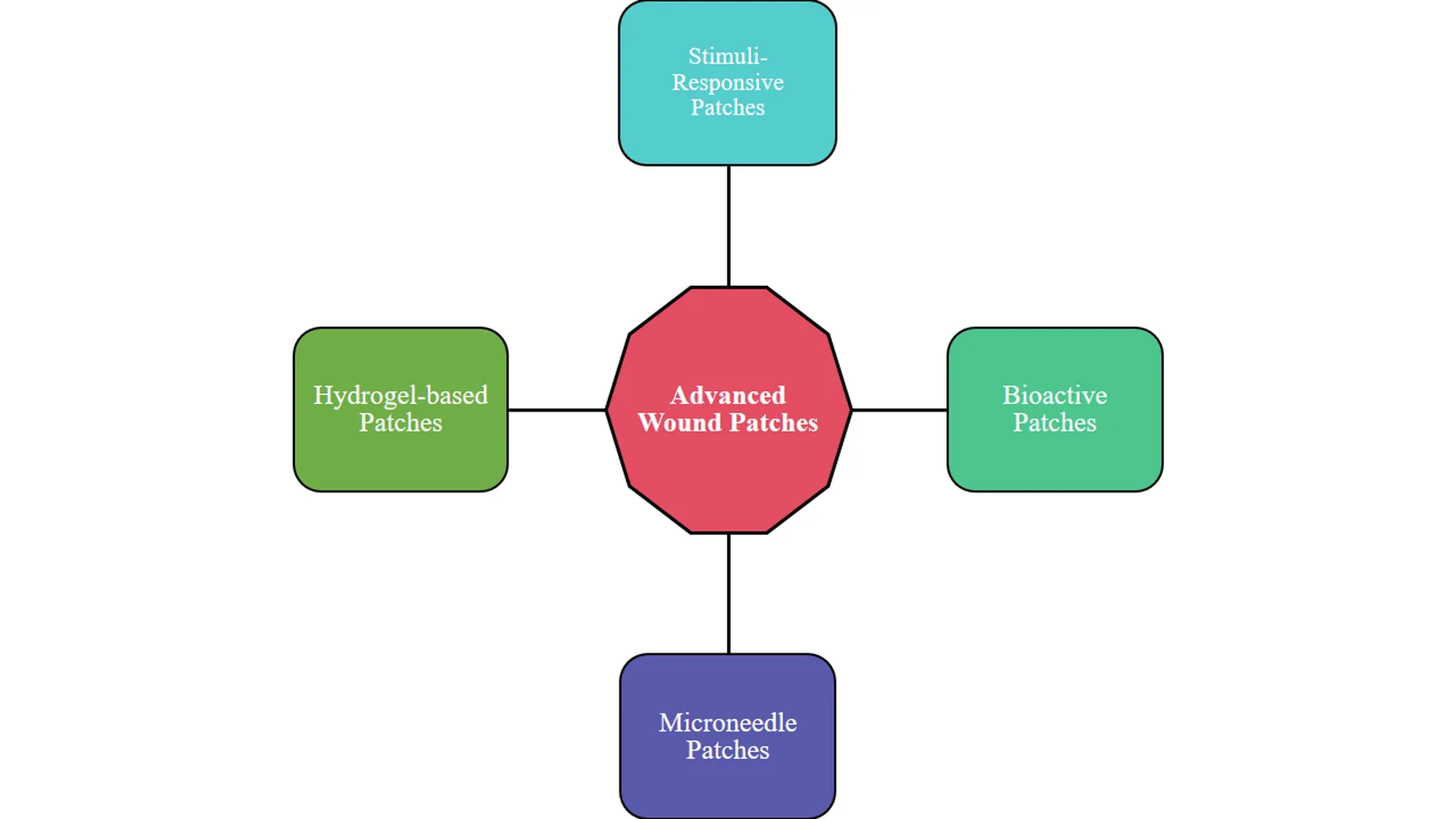
Advanced wound patches Image credit: Created by the authors using Microsoft PowerPoint (Microsoft Corporation, Redmond, WA, USA)

In the domains of biomedical engineering and regenerative medicine, smart or stimuli-responsive wound healing patches are revolutionary. These cutting-edge solutions, in contrast to conventional passive dressings, are able to recognize and actively adjust to changes in the wound microenvironment. Smart patches use responsive biomaterials, sensors, and controlled drug delivery mechanisms to give targeted and adaptive therapy that improves wound healing efficiency and patient outcomes dramatically [37]. The wound microenvironment is very dynamic, with continual biochemical and physiological changes occurring during the healing process. ROS, pH, temperature, moisture, oxygen concentration, glucose level, and enzyme activity are all important factors in determining the state of a wound. Abnormal changes in these parameters may indicate infection, persistent inflammation, or inadequate tissue regeneration in chronic wounds, such as diabetic ulcers, pressure ulcers, and venous leg ulcers. Smart wound patches are designed to detect these changes and initiate therapeutic responses accordingly. One of the most widely researched stimuli-responsive processes is pH-sensitive systems [38]. The pH of healthy skin is somewhat acidic (pH 4.5-5.5), but bacterial growth and inflammation can make chronic or infected wounds more alkaline or acidic. Under different pH settings, pH-responsive polymers can undergo structural changes, resulting in the direct release of encapsulated antibiotics, antiseptics, or anti-inflammatory medicines at the wound site. This customized distribution avoids needless antibiotic exposure and lowers the likelihood of antimicrobial resistance. Temperature-responsive patches are another essential type of smart wound dressing. Infected wounds frequently have increased local temperatures due to inflammation and microbial activity. Thermoresponsive polymers, such as poly(N-isopropylacrylamide), can alter their physical state in reaction to temperature changes, allowing for controlled medication delivery. Such devices enable prompt therapeutic action when diseased circumstances occur [39,40]. ROS-responsive wound patches are gaining popularity because excessive ROS cause oxidative stress and slow wound healing. When ROS-sensitive biomaterials detect high levels of oxidative stress, they can release antioxidants or anti-inflammatory medicines. This helps to restore redox equilibrium, minimize tissue damage, and stimulate cellular regeneration. Similarly, glucose-responsive systems are extremely useful in diabetic wound treatment. These patches detect glucose changes and provide insulin or medicinal chemicals as needed, promoting repair in diabetics. Advanced smart patches frequently include nanosensors and biosensors that can detect wound biomarkers in real time. These sensors continuously monitor wound conditions and wirelessly transmit data to healthcare providers using wearable or mobile devices. One of the biggest advantages of stimuli-responsive systems is controlled and on-demand drug distribution. Regardless of the state of the wound, conventional wound dressings often discharge drugs continuously, leading to drug waste and possible toxicity. Smart patches, on the other hand, release medications only when they are required, improving treatment precision and lowering adverse effects. Antibiotics, antimicrobial peptides, antioxidants, stem cells, growth factors, and anti-inflammatory chemicals are examples of therapeutic agents that can be used in these systems. The use of nanotechnology has enhanced the performance of smart wound patches. Nanoparticles, nanofibers, and nanogels improve drug loading efficiency, stability, antibacterial action, and tissue penetration. Multipurpose wound care platforms that can simultaneously identify, monitor, and treat wounds are also made possible by wearable technology and flexible electronics. Smart wound patches face manufacturing challenges like high production costs, long-term stability, and clinical translation despite their fascinating promise. The complex combination of electronics and biomaterials makes large-scale commercialization and regulatory approval difficult. However, it is anticipated that these limitations will be addressed by ongoing study and technological advancement. Smart wound healing systems are projected to be key components of future precision medicine and personalized healthcare approaches to chronic wound management [41,42]. The different categories of advanced wound patches, including hydrogel-based, bioactive, stimuli-responsive, and microneedle patches, are illustrated in Figure 1.

Bioactive Patches

Bioactive wound healing patches represent a significant transition from conventional passive wound dressings to cutting-edge therapeutic solutions that can actively contribute to tissue regeneration and repair. Unlike traditional dressings, which just provide physical protection and moisture retention, bioactive patches are designed to interact with biological processes around the wound site. These systems limit infection, enhance healing, and stimulate tissue regeneration through the use of medicinal compounds, cells, biomacromolecules, and nanomaterials. Hemostasis, inflammation, proliferation, angiogenesis, collagen deposition, and tissue remodeling are all part of the intricate process of wound healing. These metabolic processes are disrupted or prolonged by chronic wounds such as pressure sores, diabetic ulcers, and venous ulcers. Bioactive wound patches are intended to restore and augment these healing mechanisms by delivering therapeutic chemicals directly into the wound environment. Growth factors are a key component of bioactive patches. Growth factors that support angiogenesis, ECM production, and cell proliferation include FGF, platelet-derived growth factor (PDGF), VEGF, and epidermal growth factor (EGF) [43]. These chemicals accelerate tissue regeneration and shorten healing times when they are incorporated into wound dressings because they promote fibroblast migration, collagen production, and the formation of new blood vessels. However, the therapeutic efficacy of these growth factors may be limited by their instability in the in vitro environment, where they are susceptible to rapid degradation and loss of bioactivity, necessitating the use of appropriate stabilization and controlled-release strategies. Antimicrobial drugs are routinely used in bioactive patches to prevent or treat wound infections. Microbial colonization is particularly common in chronic wounds, slowing healing and increasing the risk of major outcomes. Because of their broad-spectrum antibacterial qualities, antibiotics, silver nanoparticles, zinc oxide nanoparticles, and copper nanoparticles are frequently used. Because they disrupt bacterial membranes, stop biofilm development, and reduce microbial resistance, silver nanoparticles are highly effective. The antioxidant, anti-inflammatory, antibacterial, and regenerative properties of plant-derived bioactive compounds have drawn more attention in recent years. Herbal extracts of aloe vera, curcumin, neem, honey, Centella asiatica, and green tea polyphenols have all been successfully used in wound patches [44]. These natural substances minimize oxidative stress, lower inflammatory cytokines, and stimulate collagen formation, resulting in better wound healing outcomes. Stem cell-loaded bioactive patches are another significant advancement in regenerative medicine. Mesenchymal stem cells (MSCs) and other progenitor cells can develop into a variety of tissues and generate growth-promoting cytokines. The incorporation of stem cells into wound dressings greatly improves tissue regeneration, angiogenesis, and immunological regulation. Similarly, exosomes and extracellular vesicles produced from stem cells are emerging as attractive alternatives due to their regenerative signaling properties. Nanotechnology plays an important role in increasing the efficacy of bioactive wound patches. Nanofibers, nanogels, liposomes, and polymeric nanoparticles improve drug encapsulation efficiency, stability, and long-term release. These nanosystems increase bioavailability and therapeutic efficacy at the wound site. Bioactive patches also help to minimize inflammation and scar formation. These systems aid in the restoration of normal tissue architecture and improved esthetic outcomes by controlling inflammatory responses and encouraging balanced collagen remodeling. Some innovative bioactive dressings integrate antibacterial, antioxidant, and angiogenic characteristics into a single multifunctional platform. Despite their promising benefits, bioactive wound patches confront a number of problems, including biomolecule instability, a short shelf life, high production costs, and potential immunogenicity. Large-scale manufacturing and long-term clinical safety require additional investigation. Nonetheless, ongoing advances in biomaterials research, tissue engineering, and nanomedicine are rapidly increasing the efficacy and practicality of these systems [45,46]. Various advanced wound patches, including hydrogel-based, stimuli-responsive, bioactive, microneedle, electronic, and electroactive systems, offer distinct benefits for wound management while presenting specific technical and clinical challenges, as illustrated in Table 2.

**Table 2:** Comparative overview of advanced wound patches ECM: extracellular matrix; ROS: reactive oxygen species

Patch type	Key features	Advantages	Limitations
Hydrogel-based patches	Moist environment, ECM mimicry	Biocompatible, promotes cell growth, enables drug delivery	Limited mechanical strength and durability [47]
Smart/stimuli-responsive patches	Respond to pH, temperature, ROS	Controlled and on-demand therapy, reduced drug wastage	Complex fabrication and high production cost [48]
Bioactive patches	Contain growth factors, nanoparticles, herbal agents	Accelerated tissue regeneration and antimicrobial action	Stability and storage challenges [49]
Microneedle patches	Microscale transdermal delivery system	High penetration efficiency and painless administration	Fabrication complexity and limited loading capacity [50]
Electronic/wearable patches	Integrated sensors and wireless monitoring	Real-time wound assessment and personalized treatment	Expensive technology and regulatory concerns [51]
Electroactive patches	Deliver electrical stimulation to wound site	Enhances cell migration, angiogenesis, and healing	Requires stable power source and conductive materials [52]

Advanced materials used in wound healing patches

The performance and functionality of advanced wound healing patches are largely determined by the materials utilized to produce them. Multifunctional systems combining biocompatibility, mechanical integrity, antibacterial activity, and stimuli responsiveness have been made possible by recent developments in biomaterials science. These materials are roughly classed as natural polymers, synthetic polymers, nanomaterials, conductive materials, and developing bioengineered systems [53,54].

Natural Polymers

Because of their great resemblance to the natural ECM, excellent biocompatibility, and biodegradability, natural polymers are crucial in the development of better wound healing patches. These biomaterials have intrinsic biological characteristics that support tissue regeneration and wound healing, and they are usually made from plants, animals, or microorganisms. They are perfect for biomedical and wound care applications due to their favorable interactions with cells and tissues. Chitosan, a polysaccharide made from chitin found in crustacean shells, is one of the most widely used natural polymers. Chitosan has excellent antibacterial, hemostatic, and bioadhesive qualities, making it ideal for wound healing applications. It can aid in clot formation, minimize microbial infection, and boost fibroblast growth. Furthermore, chitosan promotes collagen deposition and accelerates re-epithelialization, which aids in tissue regeneration. Because of its positive charge, chitosan can interact with negatively charged bacterial membranes, adding to its antibacterial properties [55]. Alginate is another major natural polymer that is often utilized in wound dressings. When calcium ions or wound exudates come into contact with alginate, a brown seaweed derivative, it creates hydrogels. These hydrogels keep the site wet and have strong fluid absorption properties, making alginate ideal for heavily oozing wounds like burns and diabetic ulcers. Furthermore, alginate dressings are non-adherent and can be removed with little damage to newly produced tissue. Collagen and gelatin are protein-based biomaterials that are frequently employed in regenerative medicine. Collagen is the principal structural protein in connective tissues, with important roles in cellular adhesion, migration, and tissue remodeling. Collagen-based dressings stimulate fibroblast proliferation and angiogenesis while decreasing inflammation. Gelatin, a denatured version of collagen, is highly biocompatible and biodegradable. It is commonly included in hydrogels, sponges, and nanofibers to aid in tissue healing and medication delivery. Hyaluronic acid is another naturally occurring polysaccharide that aids in wound healing. It is vital for tissue hydration, inflammatory control, and angiogenesis. Hyaluronic acid-based wound patches can promote cell proliferation and migration while maintaining moisture balance at the wound site. Because of its viscoelastic properties, it increases the mechanical flexibility of wound dressings. Cellulose and its derivatives are increasingly used in wound care due to their high moisture retention, mechanical stability, and biocompatibility. Bacterial cellulose, in particular, has great purity, water retention capacity, and structural strength. These characteristics make it ideal for hydrogel and nanofiber wound dressing manufacturing. Despite their obvious advantages, natural polymers have several drawbacks. Most natural biomaterials have low mechanical strength, fast breakdown, limited elasticity, and batch-to-batch variability. They may also exhibit insufficient long-term stability and tolerance to environmental stress. As a result, natural polymers are often mixed with synthetic polymers or nanomaterials to improve their structural integrity and functional performance. Recent breakthroughs in biomaterials engineering have resulted in the creation of multifunctional natural polymer-based systems that contain nanoparticles, growth factors, stem cells, and antibacterial agents. Electrospinning, 3D bioprinting, and nanotechnology have expanded the use of natural polymers in advanced wound healing patches. These hybrid systems provide increased antibacterial action, regulated medication release, and improved tissue regeneration. Overall, natural polymers remain important components in next-generation wound healing technology. Their high biological compatibility and regenerative capacity make them essential materials for developing safe, effective, and patient-friendly wound care solutions [56]. Synthetic polymers are commonly used in advanced wound healing patches because of their better mechanical qualities, customizable degradation profiles, repeatability, and ease of large-scale manufacture. Unlike natural polymers, synthetic materials may be manufactured with precise physicochemical properties, allowing researchers to customize wound dressings based on specific therapeutic needs. These polymers provide structural stability, flexibility, durability, and controlled medication release, making them critical components of current wound healing systems. PVA is one of the most extensively used synthetic polymers. PVA is a hydrophilic, biocompatible polymer capable of creating flexible, transparent hydrogels with high water retention. Its mechanical strength and film-forming ability make it suitable for wound dressings and transdermal delivery systems. PVA-based hydrogels also exhibit high oxygen permeability, which supports cellular metabolism and tissue regeneration at the wound site. PEG is another important synthetic polymer extensively used in wound care applications. PEG possesses low toxicity, excellent hydrophilicity, and minimal immunogenicity. PEG-based hydrogels are widely utilized for controlled drug delivery because they can encapsulate therapeutic agents and release them gradually over time. Additionally, PEG can be chemically modified to create stimuli-responsive systems that respond to pH, temperature, or enzymatic activity within the wound environment. Polylactic acid (PLA) and polyglycolic acid (PGA) are biodegradable synthetic polymers commonly used in tissue engineering and wound dressing fabrication. These materials degrade into non-toxic metabolites that are naturally eliminated from the body. PLA exhibits good mechanical strength and slow degradation rates, making it suitable for long-term wound healing applications. PGA, on the other hand, degrades more rapidly and is often combined with PLA to optimize degradation behavior. Polycaprolactone (PCL) is another biodegradable polymer widely utilized in nanofiber scaffolds and electrospun wound dressings. PCL possesses excellent mechanical stability, flexibility, and biocompatibility. Its slow degradation profile allows prolonged structural support during tissue regeneration. Electrospun PCL nanofibers closely mimic the ECM, promoting cell adhesion and proliferation. Polyurethane is frequently incorporated into advanced wound dressings because of its elasticity, durability, and permeability. Polyurethane films provide an effective barrier against microbial contamination while allowing oxygen exchange and moisture retention. These properties make polyurethane suitable for burns, surgical wounds, and chronic ulcers. Synthetic polymers offer several significant advantages over natural biomaterials. They exhibit consistent quality, controllable porosity, tunable mechanical properties, and adjustable degradation kinetics. Researchers can modify polymer composition, molecular weight, and crosslinking density to optimize wound healing performance. Furthermore, synthetic polymers are compatible with advanced fabrication techniques such as electrospinning, 3D printing, and microfabrication. Despite these benefits, synthetic polymers generally lack intrinsic biological activity. Unlike natural polymers, they do not naturally support cell signaling, tissue regeneration, or antimicrobial functions. Some synthetic materials may also induce inflammatory reactions or exhibit poor cell adhesion if not properly functionalized. Therefore, synthetic polymers are often blended with natural polymers, bioactive molecules, or nanoparticles to improve cellular interactions and biological performance. Recent advancements in polymer science have enabled the development of multifunctional synthetic polymer systems capable of stimuli-responsive drug delivery, self-healing behavior, and electrical conductivity [57-62]. Smart polymeric systems that respond to wound pH, temperature, or oxidative stress are becoming increasingly important in personalized wound care. Overall, synthetic polymers play a vital role in advanced wound healing technologies by providing mechanical reliability, structural support, and controlled therapeutic delivery (Table 3). Their versatility and engineering flexibility continue to drive innovation in the design of next-generation wound healing patches [63,64].

**Table 3:** Advanced materials and their roles in wound healing patches PEG: polyethylene glycol

Material type	Examples	Key properties	Role in wound healing patches
Foam materials	Polyurethane foam	Highly absorbent and cushioning	Absorbs wound exudates and protects wound from trauma [65]
Film-based materials	Polyurethane films, silicone films	Transparent, flexible, semi-permeable	Acts as a protective barrier against microbes [66]
Hydrocolloids	Gelatin, pectin, carboxymethyl cellulose	Gel-forming and adhesive	Creates moist healing environment and supports autolytic debridement [67]
Bioactive polymers	Chitosan, hyaluronic acid, collagen	Biodegradable and bioactive	Stimulates tissue regeneration and cell proliferation [68]
Nanocomposite materials	Silver nanoparticles, zinc oxide nanoparticles	Antimicrobial and anti-inflammatory	Prevents infection and accelerates healing [69]
Smart responsive materials	Thermoresponsive polymers, pH-sensitive materials	Respond to environmental stimuli	Enables controlled drug release and wound monitoring [70]
Electroconductive materials	Graphene, MXenes, conductive polymers	Electrical conductivity	Enhances cell signaling and tissue regeneration [71]
Biopolymer-based materials	Cellulose, silk fibroin, dextran	Natural origin and eco-friendly	Provides a scaffold for tissue repair [72]
Antimicrobial-loaded materials	Antibiotic-loaded patches, Herbal extract-loaded polymers	Antibacterial and anti-inflammatory	Reduces microbial contamination and infection [73]
3D printed materials	Bioinks containing alginate, gelatin, PEG	Customizable architecture	Produces patient-specific wound dressings [74]

Nanomaterials

By greatly improving the therapeutic, antibacterial, and regenerative properties of sophisticated wound patches, nanomaterials have transformed the field of wound healing. Nanomaterials have special physicochemical characteristics that are very helpful in wound care because of their nanoscale size and remarkably high surface area-to-volume ratio. Targeted medication delivery, increased antibacterial activity, better tissue regeneration, and controlled release of bioactive substances have all been made possible by their integration into wound healing systems. Silver nanoparticles (AgNPs) are one of the nanomaterials that have been investigated the most [75]. Strong broad-spectrum antibacterial action against bacteria, fungi, and diseases resistant to many drugs is exhibited by silver nanoparticles. Their antibacterial method includes interfering with DNA replication, producing ROS, and rupturing microbial cell membranes. To stop wound infection and biofilm formation, AgNPs are frequently used in hydrogel dressings, nanofiber scaffolds, and composite wound patches. Additionally, silver nanoparticles exhibit anti-inflammatory effects that further support wound healing [76,77]. Zinc oxide nanoparticles are another important class of nanomaterials used in wound care. These nanoparticles demonstrate antimicrobial, antioxidant, and anti-inflammatory properties while also promoting cell proliferation and angiogenesis. Zinc oxide nanoparticles are very useful for managing chronic wounds since zinc is crucial for collagen formation and tissue remodeling. Due to their superior stability, biocompatibility, and capacity to improve cellular interactions, gold nanoparticles have drawn more and more attention [78]. Gold nanoparticles can be used in smart wound patches as biosensing elements, photothermal agents, and medication transporters. For targeted delivery applications, their surface can be readily functionalized with growth factors, medicinal compounds, or peptides. Because of their high loading capacity and adjustable pore shapes, silica nanoparticles and mesoporous silica nanostructures are widely used for controlled drug delivery. These systems enable the targeted and prolonged release of growth hormones, anti-inflammatory drugs, and antibiotics right at the site of the wound. In a similar vein, liposomes and polymeric nanoparticles are frequently used to enhance the stability, penetration, and bioavailability of drugs. One of the most promising nanotechnology-based wound healing technologies is electrospun nanofibers. Polymers including PCL, PLA, gelatin, and chitosan are used to create nanofibrous scaffolds that closely resemble the ECM's structure. Large surface areas for drug loading are provided by their porous nature, which facilitates oxygen exchange, nutrient delivery, and cellular penetration [79]. Electrospun wound dressings can also incorporate antimicrobial agents, nanoparticles, and bioactive compounds for multifunctional therapy. Drug delivery systems that are stimuli-responsive and regulated greatly benefit from the use of nanomaterials. It is possible to create nanoparticles to release therapeutic compounds in reaction to oxidative stress, pH variations, temperature changes, or enzymes. Such intelligent delivery systems improve therapeutic precision while minimizing systemic side effects and drug resistance. Because of their mechanical strength, electrical conductivity, and antibacterial qualities, carbon-based nanomaterials like graphene oxide and carbon nanotubes are being investigated more and more for use in wound healing applications. These materials are particularly helpful in bioelectronic systems and electroactive wound dressings that use electrical signals to promote tissue regeneration [80]. Despite their remarkable advantages, nanomaterials also present several challenges. Concerns regarding cytotoxicity, oxidative stress, long-term accumulation, and environmental safety remain significant barriers to clinical translation. The interaction of nanoparticles with healthy tissues and immune systems requires careful evaluation to ensure patient safety. Furthermore, large-scale production and regulatory approval of nanomaterial-based wound dressings remain complex processes. However, the safety, effectiveness, and usefulness of nanomaterial-integrated wound healing patches are still being improved by continuous developments in nanotechnology and biomaterials science. It is anticipated that these systems will be essential to the development of regenerative therapies, precision medicine, and individualized wound care [81,82].

Key material design considerations

Key Material Design Considerations in Advanced Wound Healing Patches

The careful selection and engineering of biomaterials that can satisfy the complex physiological requirements of the wound healing process is crucial to the successful development of improved wound healing patches. Contemporary wound dressings are multifunctional therapeutic platforms that actively promote tissue regeneration, prevent infection, manage inflammation, and enable the regulated distribution of therapeutic medications. They are more than just passive protective covers. To achieve these goals, the materials used in wound healing patches must meet a number of important design criteria, including biocompatibility, moisture management, mechanical stability, antibacterial activity, controlled medication release, and stimuli responsiveness. The integration of all of these features into a single multifunctional platform is one of the most difficult issues and research objectives in contemporary wound care science [83,84]. 

Biocompatibility and Non-toxicity

One of the most crucial requirements for any material used to heal wounds is biocompatibility. The materials used in wound dressings must be devoid of toxicity, irritation, allergic reactions, and adverse immunological responses since they come into direct contact with damaged tissues for prolonged periods of time. While avoiding inflammation and tissue necrosis, an ideal wound patch should support regular cellular processes like adhesion, proliferation, migration, and differentiation. Because of their biological compatibility and structural resemblance to the ECM, natural polymers like chitosan, collagen, gelatin, alginate, and hyaluronic acid are frequently utilized. These substances support tissue regeneration and aid in cell communication. Because of their controlled physicochemical properties and low immunogenicity, synthetic polymers like PEG and PVA are also used. Synthetic materials often require surface modification or blending with bioactive compounds to maximize cell-material interactions [84,85]. Non-toxicity is especially critical in nanomaterial-based wound patches. Although they have conductive and antimicrobial properties, nanoparticles like carbon nanotubes, graphene oxide, zinc oxide, and silver can be hazardous in high concentrations. Thus, optimizing nanoparticle dosage and ensuring long-term biosafety are critical for clinical applications. Furthermore, wound dressing materials must not produce toxic breakdown products during biodegradation. Biodegradable polymers should degrade into nontoxic metabolites that can be safely removed by the body. Therefore, before advanced wound patches may be used in clinical settings, extensive in vitro and in vivo biocompatibility research is needed. Permeability and moisture retention. Effective healing depends on keeping the wound environment appropriately moist. Moisture retention stimulates epithelialization, collagen synthesis, angiogenesis, and cell migration while inhibiting scar formation and tissue drying. Excessively dry wounds slow healing and may cause tissue necrosis, whereas excessive moisture causes maceration and bacterial development. As a result, modern wound patches must provide a balanced moisture environment. Hydrogels are excellent at moisture retention due to their high water content and hydrophilic nature. Materials like alginate and hyaluronic acid can absorb wound exudates while maintaining proper hydration. Semi-permeable coatings and porous nanofiber scaffolds promote oxygen exchange while minimizing fluid loss and microbial contamination [86]. Another significant factor to consider is gas permeability, which includes oxygen and carbon dioxide. Oxygen is required for cellular metabolism, angiogenesis, and collagen formation during wound healing. Advanced dressings should therefore promote gas exchange while providing a protective barrier against microorganisms and environmental pollutants [87,88]. The porosity and microstructure of wound dressing materials have a significant impact on moisture balance and permeability. Electrospinning produces highly porous nanofibrous scaffolds that may efficiently regulate fluid absorption and oxygen transport. Such structural optimization promotes nutrient exchange and tissue regeneration.

Mechanical Strength and Flexibility

Wound healing patches must be strong enough to maintain structural integrity during application and healing. Dressings applied to joints or highly mobile body regions must be flexible, elastic, and resistant to deformation [89]. Weak or brittle materials may break during handling or provide insufficient wound protection. Synthetic polymers including polycaprolactone, polylactic acid, and polyurethane are frequently used to improve mechanical qualities. These materials offer tensile strength, flexibility, and durability. Flexibility and conformability are equally crucial because wound surfaces are frequently uneven and dynamic. A flexible wound patch may conform to the wound contour, resulting in intimate contact and better therapeutic efficacy. Soft and elastic fabrics also enhance patient comfort and alleviate pain when moving. Mechanical qualities have an extra influence on cellular behavior. Thus, improving the mechanical properties of wound dressings is critical not only for structural performance but also for biological activity [90].

Antimicrobial Activity

Infection is a common consequence of chronic and acute wounds. Microbial colonization slows healing, causes inflammation, and can lead to serious systemic infections. As a result, enhanced wound healing patches should have strong antimicrobial characteristics to inhibit bacterial growth and biofilm formation [91].

Chitosan is frequently utilized due to its innate antibacterial properties. It damages microbial cell membranes and prevents bacterial multiplication. Because of their broad-spectrum effectiveness against bacteria, fungi, and multidrug-resistant illnesses, silver nanoparticles are among the most thoroughly studied antimicrobial agents. To enhance infection control, wound dressings also contain antimicrobial peptides, copper nanoparticles, and zinc oxide nanoparticles.

**Figure 2:**
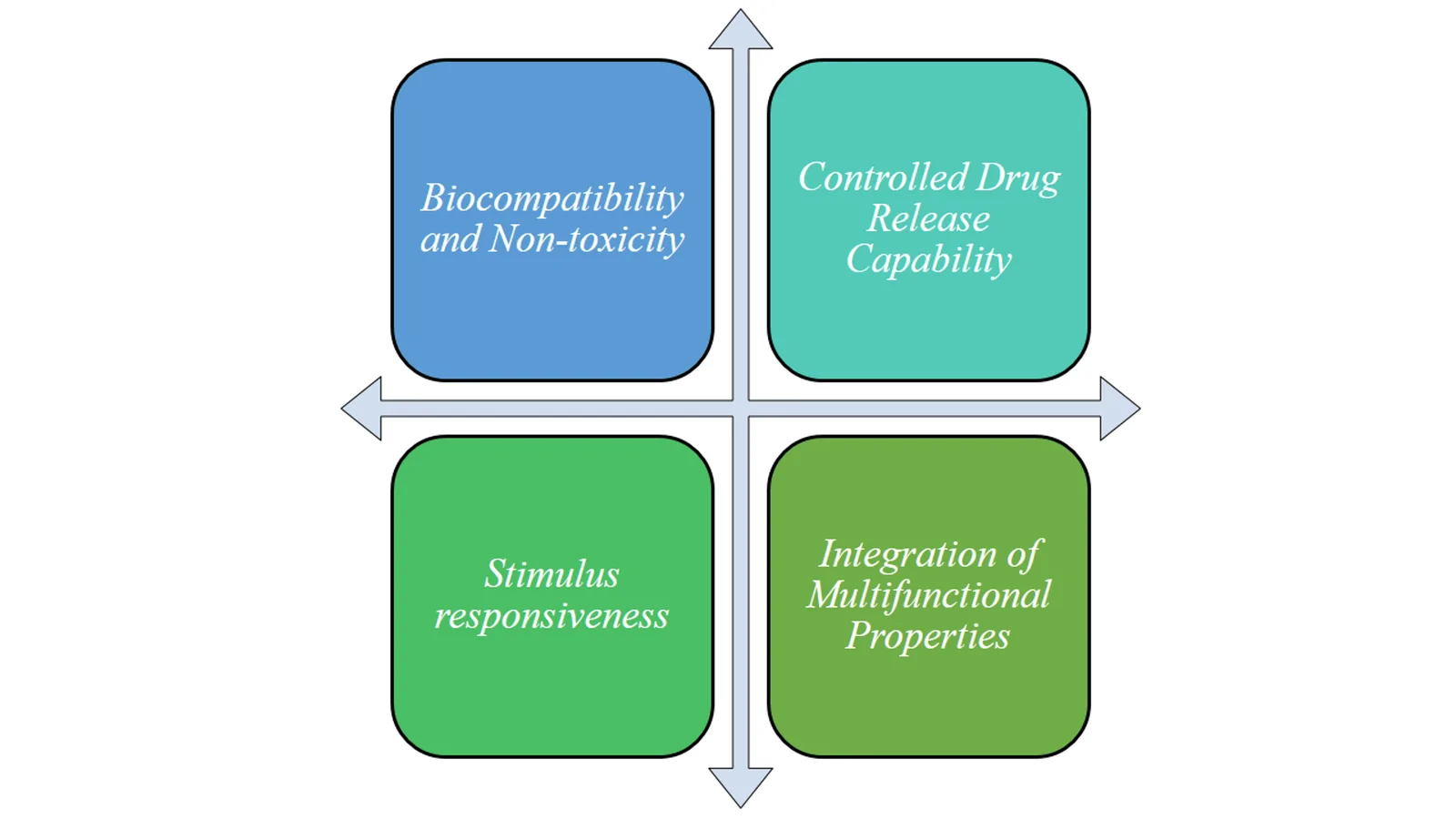
Key material design considerations in advanced wound healing patches Image credit: Created by the authors using Microsoft PowerPoint (Microsoft Corporation, Redmond, WA, USA)

Curcumin, aloe vera, honey, neem, and essential oils are all plant-derived bioactive substances that give further antibacterial and anti-inflammatory properties. Combining antimicrobial drugs can increase efficacy while reducing bacterial resistance. An optimal antimicrobial wound patch should destroy harmful germs while preserving healthy host cells. Controlled release techniques are especially critical since excessive antimicrobial exposure can cause cytotoxicity and inhibit tissue regeneration. As a result, balancing antibacterial efficacy with cellular compatibility remains an important research priority [92]. The essential material design requirements for advanced wound healing patches, including biocompatibility, non-toxicity, controlled drug release capability, stimulus responsiveness, and multifunctional properties, are illustrated in Figure 2.

Controlled Drug Release Capability

Improved wound healing systems must have controlled and localized medication distribution. Repeated drug application is often required with conventional dressings, which leads to inconsistent therapeutic concentrations and decreased patient compliance. On the other hand, medicinal compounds can be encapsulated in new wound patches and released gradually over time. While reducing systemic side effects and medication waste, controlled drug release improves therapeutic efficacy. Antibiotics, growth factors, anti-inflammatory medicines, antioxidants, stem cells, peptides, and nucleic acids are all common therapeutic agents used in wound dressings [93].

Hydrogels, nanofibers, liposomes, and nanoparticle-based carriers are commonly used for controlled drug delivery. Polymer composition, crosslinking density, degradation rate, and nanostructure design can all affect the release kinetics. Sustained medication release keeps therapeutic concentrations at the wound site for longer times, decreasing the need for dressing changes. Growth factor-loaded dressings stimulate angiogenesis and collagen deposition, whereas antioxidant systems minimize ROS and tissue damage. Smart delivery methods that adapt to environmental cues improve treatment precision [94,95].

Stimulus Responsiveness

Stimuli-responsive wound patches are among the most advanced breakthroughs in wound care technology. These intelligent systems can sense changes in the wound microenvironment and react by releasing therapeutic substances or modifying their physical qualities. Typical stimuli include pH, temperature, ROS, glucose concentration, and enzymatic activity. Infected wounds frequently have acidic pH and higher temperature, whereas chronic wounds have high oxidative stress and aberrant glucose levels. Responsive materials can use these pathological changes to cause site-specific medication release [96]. pH-sensitive polymers are commonly employed to release antibiotics under the acidic conditions associated with illness. When the temperature changes, thermoresponsive hydrogels can modify how they swell. When oxidative stress levels rise, ROS-responsive systems respond by releasing antioxidants. Glucose-responsive materials are extremely useful for diabetic wound care. Stimuli-responsive wound patches allow for on-demand therapy, reducing unnecessary drug exposure while improving treatment precision. The integration of biosensors and wearable electronics improves their functionality by allowing real-time monitoring and autonomous therapeutic feedback [97].

Integration of Multifunctional Properties

One of the most difficult issues in advanced wound patch creation is combining all necessary features into a single multifunctional solution. It is extremely difficult to achieve optimal biocompatibility, antibacterial activity, mechanical stability, moisture regulation, controlled medication release, and stimuli response all at the same time. Hybrid materials and composite systems are increasingly being used to overcome this difficulty. Natural polymers are coupled with synthetic polymers, nanoparticles, conductive materials, and bioactive substances to build synergistic multifunctional platforms [98]. Examples of integrated systems include nanocomposite hydrogels, electroactive scaffolds, and bioengineered matrices. Emerging technologies including electrospinning, 3D bioprinting, microfabrication, and nanotechnology are hastening the development of next-generation wound healing patches. Artificial intelligence and biosensor integration are enhancing individualized and precise wound treatment [99]. Overall, material design concerns are critical to the development of intelligent wound healing systems. Continued interdisciplinary research in biomaterials science, nanotechnology, tissue engineering, and bioelectronics will be required to develop safe, effective, and clinically applicable wound healing patches capable of addressing the expanding worldwide burden of chronic wounds [100].

Key technological innovations in wound healing patches

The convergence of biomaterials research, nanotechnology, electronics, and digital health has propelled recent advancements in wound healing patches. These advancements have converted traditional wound dressings into multifunctional, intelligent platforms capable of real-time monitoring, targeted treatments, and personalised care. The following important technological advancements shape the present landscape of next-generation wound healing systems [101,102].

Intelligent Sensing and Real-Time Monitoring

Smart sensing technologies have transformed modern wound management by allowing for continuous, noninvasive monitoring of the wound microenvironment. Traditional wound assessment procedures frequently rely on visual inspection and periodic clinical evaluation, which may miss early indicators of infection or delayed healing. By offering real-time data on biochemical and physiological changes in the wound bed, the incorporation of biosensors into wound healing patches goes beyond these limitations. These cutting-edge tools are particularly helpful in chronic wounds when early intervention is crucial to preventing complications, such as diabetic ulcers, pressure ulcers, and burn injuries [103]. Wound pH is one of the most routinely observed characteristics. Smart wound patches with pH-sensitive dyes, electrochemical sensors, or fluorescence-based indicators can detect these changes and provide visual or electronic feedback on wound condition. Temperature sensing is another important feature, as high local temperatures frequently suggest inflammation or bacterial infection. Flexible thermal sensors integrated into wound dressings allow for continuous temperature tracking without disturbing the wound surface. In addition to pH and temperature, contemporary biosensors may measure oxygen, glucose, uric acid, lactate, and inflammatory cytokines. Oxygen monitoring is especially crucial since tissue hypoxia slows angiogenesis and cellular proliferation. Since high glucose levels can impede healing and raise the risk of infection, glucose sensors are very helpful for managing diabetic wounds. Multiplexed sensing platforms capable of monitoring many biomarkers at the same time are being developed to provide a thorough wound assessment. Flexible electronics and elastic conductive materials play an important role in wearable sensing devices. Materials like graphene, gold nanoparticles, conductive polymers, and carbon nanotubes enable the creation of highly sensitive biosensors that conform to uneven wound surfaces. Wound data can be remotely transferred to doctors or mobile devices using wireless communication technologies like Bluetooth and near-field communication (NFC). Such devices enable telemedicine and reduce the number of hospital visits. Overall, smart sensing wound patches represent a significant step forward in individualized and precise wound treatment. These devices considerably improve treatment results and patient quality of life by allowing for early diagnosis of problems and ongoing monitoring of healing progression [104].

Controlled and Targeted Drug Delivery Systems

Among the most important developments in improved wound healing patches are controlled and targeted medicine delivery systems. Topical medications must often be applied repeatedly when using conventional wound dressings, which can lead to uneven drug concentrations, low patient compliance, and possible systemic side effects. By enabling the local, continuous release of therapeutic chemicals in response to stimuli at the wound site, advanced wound patches go beyond these limitations. Numerous therapeutic substances, including antibiotics, antifungals, anti-inflammatory drugs, antioxidants, growth factors, stem cells, and nucleic acids, are found in contemporary wound patches [105]. The major goal is to maintain adequate therapeutic concentrations over time while reducing unwanted exposure to healthy tissues. Controlled drug distribution also lowers the need for dressing replacement, lowering wound disturbance and discomfort.

Stimuli-responsive systems are especially useful in focused wound therapy. ROS, pH, temperature, glucose, and bacterial enzymes are examples of environmental cues that these systems react to. Infected wounds, for example, tend to have an acidic pH and high ROS levels. pH-sensitive hydrogels or nanoparticles can release antibiotics only in acidic environments, ensuring site-specific treatment while minimizing antibiotic resistance. Similarly, ROS-responsive systems produce antioxidants or anti-inflammatory substances only when oxidative stress levels rise. Nanotechnology has dramatically improved medicine delivery performance in wound patches. Nanoparticles, liposomes, dendrimers, micelles, and nanofibers all enhance drug stability, penetration, and bioavailability. Electrospun nanofibrous scaffolds are particularly beneficial because their large surface area allows for efficient drug loading and sustained release. Silver nanoparticles and zinc oxide nanoparticles have intrinsic antibacterial activity, which improves treatment efficacy [106]. Hydrogels are another popular platform for regulated distribution because of their porous structure and high water content. Drug release kinetics can be altered by changing the polymer composition, crosslinking density, or degradation rate. Multi-layered and dual-drug delivery systems have also been created to release different therapeutic agents in a sequential manner based on the wound healing stage. Targeted drug delivery methods considerably improve wound healing results by increasing angiogenesis, decreasing infection, regulating inflammation, and speeding up tissue regeneration. As research progresses, the integration of smart biomaterials, nanotechnology, and bioresponsive polymers is predicted to improve precision wound therapy and enable the development of fully autonomous therapeutic wound care platforms [107].

Theranostic Wound Patch System

Theranostic wound patch systems are a next-generation technique that integrates diagnostic and therapeutic capabilities on a single integrated platform. Unlike traditional wound dressings, which provide passive protection, theranostic patches actively monitor wound conditions and administer tailored therapy in reaction to anomalies. This closed-loop method has the potential to significantly enhance results in chronic and challenging-to-heal wounds by enabling autonomous wound management [108,109].

Theranostic systems' diagnostic components often include biosensors that may identify biomarkers linked with infection, inflammation, hypoxia, or delayed healing. Continuously measured parameters include pH, temperature, oxygen concentration, glucose levels, bacterial toxins, and inflammatory cytokines. Changes in these biomarkers reveal important information about wound development and associated consequences. Elevated pH and temperature, for example, may indicate bacterial colonization, but low oxygen levels indicate poor vascularization.

When aberrant conditions are detected, the therapeutic component is turned on automatically. Drug-loaded hydrogels, nanoparticles, microneedles, and electrospun nanofibers provide antibiotics, anti-inflammatory medicines, antioxidants, and growth hormones in a regulated manner. This responsive drug delivery system provides prompt intervention while reducing unnecessary drug exposure. In some systems, electrical stimulation or photothermal therapy is used to boost tissue regeneration and antimicrobial activity [110].

In theranostic systems, nanotechnology is crucial. Multifunctional nanoparticles can be utilized simultaneously for drug administration, sensing, and imaging. For example, fluorescent nanoparticles can detect bacterial toxins while also releasing antimicrobial compounds. Similarly, conductive materials, such as graphene and gold nanoparticles, improve signal transmission and sensor performance. Wearable electronics and wireless connectivity improve theranostic capabilities. Healthcare professionals can get real-time wound data via cloud-based systems or cellphones, enabling remote monitoring and customized treatment adjustments. Such connectedness is especially useful for elderly patients and people with chronic conditions that need long-term wound care. Despite great development, there are still several hurdles in the clinical translation of theranostic wound patches. These include device complexity, manufacturing costs, long-term stability, power supply needs, and regulatory approval. Nonetheless, theranostic systems represent a significant paradigm change toward intelligent, adaptive, and individualized wound management, with huge promise for lowering healthcare costs and improving patient outcomes [111,112].

Integration of Wearable Electronics with Wireless Communication

Wound healing patches are now cognitive healthcare platforms with real-time monitoring and remote physician administration thanks to wearable electronics and wireless connection technology. Traditional wound care approaches frequently include regular hospital visits and physical wound assessments, which can be uncomfortable, costly, and unsuccessful for chronic wound patients. Wearable electronic wound patches address these constraints by allowing for real-time data gathering and wireless transfer of wound-related information [113]. Flexible circuits, microcontrollers, biosensors, antennas, and communication modules are all integrated into modern wearable wound patches made of soft, biocompatible materials. These systems are intended to adhere to the skin surface without impeding patient movement. Flexible and stretchable electronic components made from conductive polymers, graphene, carbon nanotubes, and metallic nanowires are mechanically compatible with biological tissues. Wound data can be continuously transmitted to cellphones, computers, or cloud-based healthcare systems thanks to wireless communication technologies including Bluetooth, NFC, Wi-Fi, and RFID. Wound parameters like temperature, pH, moisture, oxygen saturation, and glucose level can be remotely monitored by clinicians. By eliminating the need for regular dressing removal or hospital visits, this capability promotes telemedicine. Remote monitoring has various clinical advantages. Early diagnosis of infection, inflammation, or delayed healing allows for immediate therapeutic action, which reduces complications and hospitalization rates. Continuous data gathering also allows for longitudinal investigation of wound healing patterns, resulting in tailored treatment optimization. Furthermore, wearable solutions increase patient compliance and comfort by reducing unwanted wound manipulation [114]. Advanced wearable patches may also include drug delivery devices and feedback-controlled therapy. For example, if sensors detect increased temperatures or acidic pH levels associated with infection, the gadget can instantly release antibiotics. Such closed-loop technologies are a significant step towards autonomous wound management. Energy supply is an important consideration for wearable electronic devices. Researchers are becoming interested in Self-powered devices that collect energy from human movement, heat, or biological interactions, including biofuel cells, piezoelectric materials, and triboelectric nanogenerators. These technologies lessen dependency on traditional batteries while increasing device sustainability. Although great progress has been made, device durability, sensor calibration, data security, manufacturing scalability, and regulatory approval remain major impediments to commercialization. Nonetheless, wearable electronics and wireless communication technologies are projected to be critical components of future precision wound care and digital healthcare systems [115]. 

Electrostimulation and Bioelectronic Therapy

Electrical stimulation has emerged as an extremely promising method for speeding up wound healing and tissue regeneration. Natural skin contains endogenous electric fields that direct vital healing processes like angiogenesis, collagen production, cell migration, and proliferation. Chronic wounds frequently disrupt these bioelectric impulses, resulting in poor healing. Advanced bioelectronic wound patches seek to restore or augment electrical cues that stimulate tissue recovery [116,117].

3D Printing and Biofabrication Technology

Conductive materials utilized in contemporary electroactive wound patches include polypyrrole, polyaniline, graphene, carbon nanotubes, and metallic nanoparticles. These materials provide excellent electrical conductivity while remaining flexible and biocompatible. Conductive hydrogels are very useful because they combine electrical performance, moisture retention, and tissue compatibility. In wound therapy, many kinds of electrical stimulation are used, such as direct current, pulsed current, alternating current, and microcurrent stimulation. The ideal electrical parameters are determined by the type of wound, its severity, and the therapeutic goals. Controlled transmission of electrical signals improves therapeutic precision while minimizing tissue harm.

Recent advancements have centered on self-powered bioelectronic systems [118]. Triboelectric nanogenerators and piezoelectric materials can convert the mechanical energy generated by bodily movement or respiration into electrical stimulation. These self-sustaining devices reduce the need for external power sources, increasing patient convenience. Some advanced patches additionally include biosensors that monitor wound conditions and change electrical stimulation accordingly.

For chronic wounds where inadequate blood circulation and cellular activity hinder healing, such as diabetic foot ulcers, venous ulcers, and pressure sores, electrical stimulation is particularly beneficial. Clinical investigations have shown that electrotherapy-based devices expedite wound closure, improve angiogenesis, and lower infection rates. Despite the promising results, various obstacles remain, including long-term safety, electrical dosage optimization, device durability, and large-scale production. Additional clinical investigations and regulatory validation are required before general implementation. Nonetheless, bioelectronic wound patches are a highly novel and promising approach to regenerative medicine and enhanced wound treatment [119].

3D printing and biofabrication technologies have transformed the development of sophisticated wound healing patches, allowing for exact control over structure, composition, and functionality. Conventional fabrication methods frequently fail to produce complicated designs that accurately mirror biological tissue organization. On the other hand, fully personalized wound dressings based on specific patient needs and wound characteristics can be made thanks to 3D printing.

Common 3D printing methods used in wound engineering include extrusion-based printing, inkjet printing, stereolithography, and electrohydrodynamic printing. These techniques make it possible to precisely deposit biomaterials, cells, growth agents, and nanoparticles layer by layer. The resultant constructions have precisely regulated porosity, mechanical strength, degradation behavior, and drug release characteristics. Natural or synthetic hydrogels like alginate, gelatin, collagen, hyaluronic acid, PEG, or fibrin are frequently used to create wound bioinks. These substances encourage the adhesion, proliferation, and differentiation of cells. To promote tissue regeneration and vascularization, printed structures can incorporate stem cells, fibroblasts, keratinocytes, endothelial cells, and bioactive substances. The ability of 3D printing to produce patient-specific wound patches based on wound geometry and severity is a key benefit. To create precisely fitting dressings, computer-aided design tools can be used in conjunction with advanced imaging techniques such as CT scanning and 3D wound mapping. This kind of tailoring enhances wound coverage, removes dead space, and boosts healing effectiveness [120].

AI and Data-Driven Wound Care

Artificial intelligence (AI) and data-driven technologies are changing wound management by allowing for predictive analytics, automated diagnostics, and individualized treatment plans. Chronic wound care creates massive volumes of clinical, imaging, and sensor-based data, which can be challenging to evaluate with traditional methods. Deep learning algorithms can examine wound pictures to determine wound size, tissue composition, exudate levels, necrosis, granulation tissue, and infection status. Automated image analysis decreases subjectivity associated with manual assessment while improving diagnostic consistency. Additionally, AI algorithms are able to predict patterns of wound healing and identify patients who are most vulnerable to complications or delayed recovery. The combination of AI and smart wound patches improves monitoring capabilities. Biosensors embedded in patches collect continuous data on pH, temperature, glucose levels, oxygen concentration, and inflammatory indicators. This data is processed by machine learning algorithms to detect small changes that may indicate infection, inflammation, or poor healing. Predictive models can make individualized therapy recommendations based on real-time wound conditions. AI-powered wound care solutions also enable telemedicine and remote patient management. Cloud-based solutions allow doctors to access wound data from remote locations, track healing progress, and alter treatment regimens without having to make regular in-person visits. This method is very useful for the elderly, diabetic patients, and those living in distant places. Natural language processing and electronic health record integration enable AI systems to compile clinical history, laboratory results, imaging data, and sensor outputs into full patient profiles. Such multimodal analysis increases treatment accuracy and complements precision medicine approaches in wound care. Recent research has also looked at autonomous wound care systems that use AI, biosensors, and responsive medicine delivery techniques. When problems are discovered in these systems, AI algorithms scan wound data and automatically release drugs or stimulate electrically [121,122]. These closed-loop platforms reflect the future of sophisticated wound treatment. Despite promising developments, several challenges remain, including data privacy, algorithm bias, clinical validation, interoperability, and regulatory approval. Large, high-quality datasets are required to train accurate machine learning models. Nonetheless, AI-powered wound care is likely to increase diagnostic accuracy, treatment efficiency, and healthcare accessibility in the future years [123].

Clinical applications for advanced wound healing patches

Advanced wound healing patches have shown promising therapeutic results for a variety of acute and chronic wound diseases. These patches address important shortcomings of conventional wound care, including infection control, delayed healing, and a lack of real-time monitoring, by fusing biomaterials, drug delivery systems, and smart sensing technologies. As explained below, they can be used in a variety of clinical situations [124].

Diabetic Foot Ulcers

DFUs are one of the most difficult chronic wound diseases, frequently accompanied by poor angiogenesis, neuropathy, and persistent inflammation. It has been demonstrated that advanced wound patches, especially hydrogel-based and bioactive systems, efficiently preserve a moist environment, encourage the formation of granulation tissue, and enhance re-epithelialization.

Smart patches that monitor glucose levels, pH, and infection markers are extremely useful in diabetic wound treatment. Patches with growth hormones, stem cells, or antimicrobial agents promote faster healing and lower the chance of amputation [125,126].

Burn Wounds

Burn injuries necessitate specific wound care to prevent infection, control exudate, and promote controlled tissue regeneration while minimizing fibrosis and excessive scar formation. Advanced wound patches aim to accelerate healing while providing a favorable microenvironment for functional tissue repair. Advanced wound patches, such as nanofiber scaffolds and hydrogel dressings, effectively retain moisture and provide thermal insulation while also promoting cell proliferation. Bioactive and antimicrobial patches coated with silver nanoparticles or antibiotics are commonly used to prevent microbial colonization in burn sites. Conductive and electroactive patches can enhance cellular activity and angiogenesis in injured tissues, potentially speeding up the healing process [127].

Pressure Ulcers

**Figure 3:**
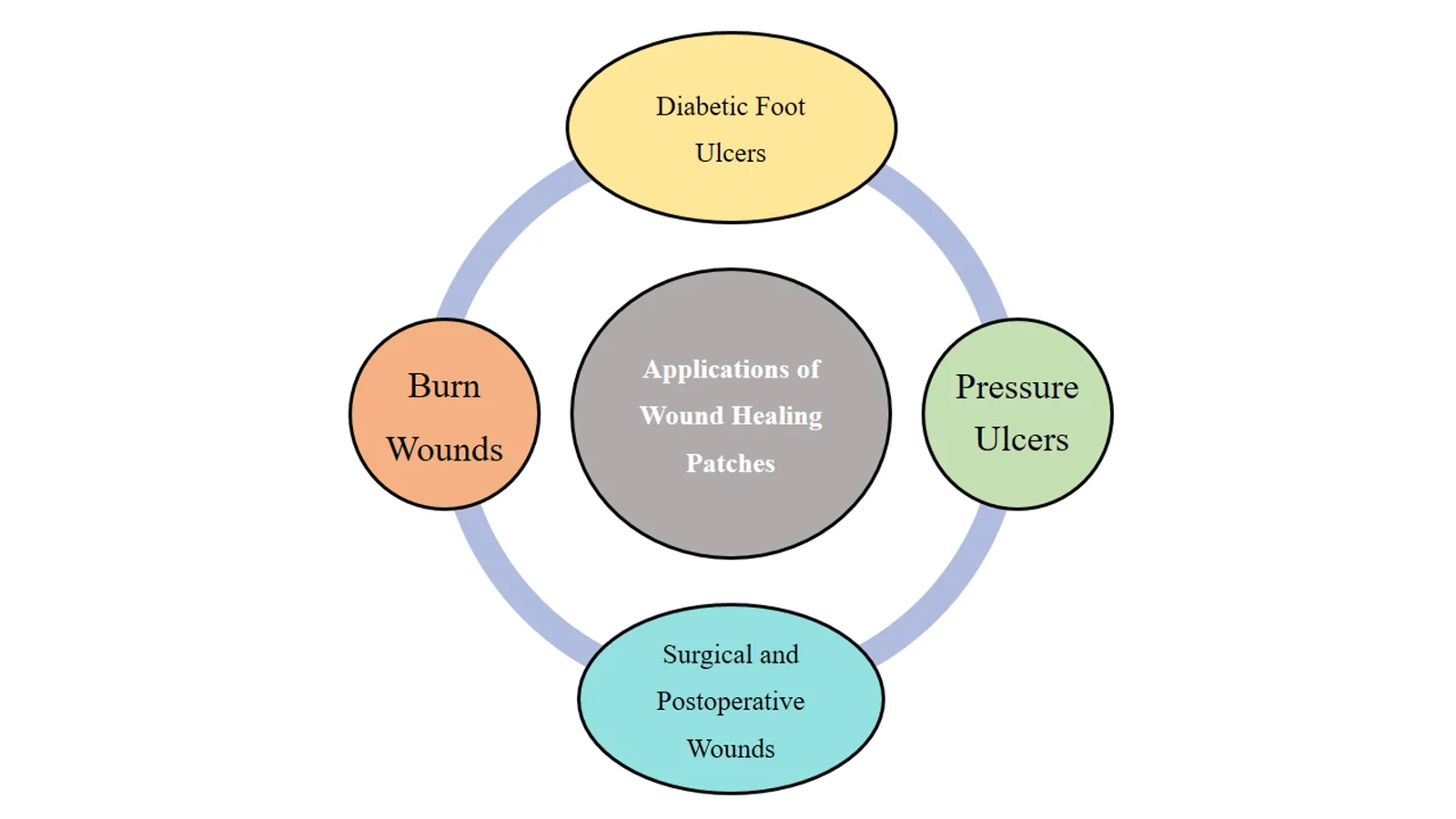
Applications of advanced wound healing patches Image credit: Created by the authors using Microsoft PowerPoint (Microsoft Corporation, Redmond, WA, USA)

Pressure ulcers, which are common in immobile or elderly individuals, are caused by prolonged pressure and restricted blood supply to the tissues. Advanced wound healing patches help to reduce these symptoms by increasing oxygen permeability, preserving hydration, and stimulating tissue regeneration [128]. Smart patches with real-time monitoring capabilities can detect early indicators of tissue damage, allowing for prompt intervention. Bioactive compounds that promote collagen production and angiogenesis can help speed up recovery from pressure injuries [129]. As depicted in Figure 3, advanced wound healing patches have found extensive applications in infection control, tissue regeneration, chronic wound management, drug delivery, and real-time wound monitoring.

Surgical and Post-operative Wounds

For post-surgical wounds to minimize the danger of infection and maximize scar formation, controlled healing conditions are necessary. Advanced wound patches create protective barriers while also providing therapeutic ingredients like anti-inflammatory medicines and growth hormones. Electronic and wearable patches offer continuous wound monitoring, enabling early diagnosis of issues like infection or dehiscence. These platforms enhance patient outcomes by promoting early intervention and lowering hospital readmission rates [130].

Chronic Non-healing Wounds

Chronic wounds, such as venous leg ulcers and ischemic wounds, are distinguished by persistent inflammation, poor vascularization, and biofilm growth. Advanced wound patches are specifically intended to solve these issues using multifunctional techniques. Stimuli-responsive and theranostic patches can detect pathogenic changes and administer targeted therapy, such as antibacterial or anti-inflammatory chemicals. Electroactive patches improve healing by promoting cellular migration and tissue regeneration. These integrated systems have demonstrated promising outcomes in overcoming the limits of traditional therapy [131,132].

Military and Traumatic Wounds

In emergency and battlefield situations, quick and effective wound care is essential. Advanced wound patches with hemostatic, antibacterial, and self-adhesive qualities offer instant protection and encourage rapid healing. Smart patches that are portable and simple to apply are crucial to trauma care because they enable on-site monitoring and rehabilitation. Self-powered and durable devices are especially useful in resource-limited areas where medical supervision is not always available [133].

Clinical benefits and outcomes

Enhanced Wound Healing and Tissue Regeneration

Advanced wound healing patches expedite the healing process by establishing an ideal milieu for tissue restoration. Modern patches maintain the proper moisture balance, increase oxygen exchange, and promote cellular growth and migration. Growth factors, stem-cell-derived compounds, and antimicrobial agents are among the bioactive components that promote ECM remodeling, collagen deposition, and angiogenesis. Systems based on hydrogel and nanofiber closely mimic the ECM found in nature, which increases keratinocyte regeneration and fibroblast activity. The danger of recurrent infections and the development of chronic wounds is reduced by quicker wound closure. These benefits are particularly crucial for burn injuries, diabetic ulcers, and pressure ulcers, where delayed healing presents a major therapeutic challenge and may lead to major complications or hospitalization [134].

Reduced Infection Rates by Antimicrobial Activity

One of the primary functions of advanced wound healing patches is to avoid infections. Modern dressings contain antimicrobial agents such as silver nanoparticles, zinc oxide nanoparticles, antimicrobial peptides, and herbal bioactive ingredients that prevent bacterial growth and biofilm formation. Smart wound patches can also detect infection-related changes, such as acidic pH or high temperature, and respond by releasing antibiotics directly to the wound site. This targeted therapy lowers microbial load while limiting systemic antibiotic exposure and resistance development. By managing infection, these patches reduce inflammation, prevent tissue necrosis, and enhance healing results. Reduced infection rates lead to fewer hospitalizations, lower healthcare expenses, and fewer invasive surgical interventions in chronic or severe wounds [135].

Reduced Need for Frequent Dressing Changes

Traditional wound dressings frequently require changing, which can disrupt freshly produced tissue and cause patient discomfort. Advanced wound healing patches are intended to provide long-term functionality through sustained medication release, better moisture retention, and structural durability. Hydrogel-based and bioengineered dressings can keep a wound in good condition for long periods of time, minimizing the need for dressing changes. This reduces stress to regenerated tissue and the possibility of contamination during dressing removal. Reduced dressing replacement reduces both nurse workload and healthcare resource use. For patients with chronic wounds or burns, fewer dressing changes increase overall treatment convenience and alleviate pain associated with recurrent wound exposure, resulting in more efficient long-term wound care [136].

Increased Patient Comfort and Compliance

Advanced wound patches are specifically intended to improve patient comfort by using flexible, lightweight, and breathable fabrics that fit tightly to the skin surface. Hydrogels have a cooling and relaxing action that relieves pain and irritation, especially in burn wounds and inflammatory skin lesions. Wearable and sticky patches enable patients to resume their everyday activities with minimal inconvenience. Reduced dressing changes and regulated therapeutic administration enhance convenience and treatment adherence. Smart patches with remote monitoring eliminate the need for frequent hospital visits, which improves the quality of life for the elderly and chronically ill patients. Improved comfort and usefulness lead to higher patient compliance, which is crucial for obtaining good wound healing outcomes and reducing treatment failure [137].

Real-Time Monitoring Enables Personalized Treatment

Continuous real-time monitoring of wound parameters like pH, temperature, oxygen levels, glucose concentration, and inflammatory biomarkers is made possible by the integration of wearable electronics and biosensors into wound healing patches. These technologies enable clinicians to continuously track the wound healing timeline by providing real-time information on infection, inflammation, and healing progression. Personalized treatment tactics can then be altered based on the patient's wound status, increasing therapeutic precision and effectiveness. Some smart patches can automatically release medications in response to recognized irregularities, allowing for adaptive and closed-loop wound treatment. Remote monitoring capabilities also facilitate telemedicine and home-based care, minimizing hospital reliance. Real-time data gathering paired with artificial intelligence has the ability to predict healing results and optimize personalized wound care in future precision medicine applications [138,139].

Challenges and limitations of advanced wound healing patches

Despite substantial technological advances, the clinical translation and broad use of enhanced wound healing patches are hampered by a number of scientific, technical, regulatory, and commercial obstacles. Successfully integrating next-generation wound care systems into ordinary clinical practice requires addressing these obstacles.

Biocompatibility and Safety Issues

While many sophisticated wound patches use biocompatible materials, the addition of nanoparticles, conductive polymers, and bioactive chemicals increases the risk of cytotoxicity, immunogenicity, and long-term tissue damage. For example, an excessive accumulation of metallic nanoparticles (such as silver or zinc oxide) can cause oxidative stress and cellular toxicity. Similarly, conductive materials like graphene and carbon nanotubes, while useful for electrical signaling, pose questions about biodegradability and long-term safety. Ensuring material safety through extensive in vitro and in vivo testing remains a significant problem [140].

Limited Clinical Evidence and Translation Gaps

A major portion of improved wound healing technologies are currently in preclinical or early clinical development. Large-scale, randomized controlled trials to confirm their effectiveness and safety in a variety of patient populations have been scarce, despite encouraging results being established in lab and animal models. Commercialization is delayed, and regulatory approval is hampered by the gap between experimental research and clinical use. Standardized procedures and long-term outcome studies are necessary to establish clinical dependability [141].

High Cost and Economic Constraints

The incorporation of sophisticated materials, nanotechnology, and electronic components considerably raises the cost of wound healing patches. Smart and theranostic systems, in particular, need sophisticated fabrication techniques and expensive materials, making them less accessible to low- and middle-income populations. Adoption in healthcare systems, particularly for chronic wound management, is heavily influenced by cost-effectiveness and treatment duration [142].

Manufacturing and Scalability Issues

Advanced wound patches, particularly those including nanomaterials, microneedles, or bioengineered constructs, require complex production techniques such as electrospinning, 3D bioprinting, and microfabrication. Scaling up these procedures while ensuring consistency, reproducibility, and quality control is a significant task. Incorporating several features (e.g., sensing, medication delivery, and electrical stimulation) into a single platform might complicate manufacturing [143]. As highlighted in Table 4, overcoming challenges such as high production costs, limited clinical evidence, regulatory barriers, and scalability issues will be critical for the successful integration of advanced wound healing patches into routine clinical practice.

**Table 4:** Challenges, impacts, and potential solutions for advanced wound healing patches

Challenge/limitation	Description	Impact on wound healing	Possible solution	Examples of affected materials/systems
High production cost	Advanced materials, nanotechnology, and smart systems increase manufacturing expenses	Limits widespread clinical adoption	Development of cost-effective fabrication methods	Smart patches, nanocomposite dressings [144]
Limited mechanical strength	Some patches may tear or lose integrity during prolonged application	Reduced durability and protection	Reinforcement with composite polymers	Biopolymer films, hydrocolloids [145]
Risk of infection	Improper handling or prolonged use can still lead to microbial contamination	Delayed healing and complications	Incorporation of antimicrobial agents	Conventional and bioactive dressings [146]
Biocompatibility concerns	Certain synthetic polymers and nanoparticles may cause irritation or toxicity	Inflammation and allergic reactions	Use of safer biodegradable materials	Nanoparticle-loaded patches [147]
Complex manufacturing processes	Fabrication methods such as electrospinning and 3D printing require specialized equipment	Difficult large-scale production	Simplified and automated production systems	Electrospun and 3D-printed patches [148]
Challenges in achieving precise and sustained drug release	Maintaining sustained and precise therapeutic release remains challenging	Inconsistent therapeutic effect	Smart responsive delivery systems	Drug-loaded wound patches [149]
Short shelf life	Bioactive and biodegradable materials may degrade during storage	Reduced product effectiveness	Improved packaging and stabilization methods	Collagen and herbal-based patches [150]
Moisture imbalance	Excessive moisture retention can cause maceration, while insufficient moisture delays healing	Poor wound environment management	Optimization of absorbent capacity	Foam and film dressings [151]
Regulatory and approval barriers	Advanced wound patches require extensive clinical testing and regulatory validation	Delayed market availability	Streamlined regulatory pathways	Smart and nanotechnology-based systems [152]
Limited accessibility	High cost and limited availability reduce use in low-resource settings	Restricted patient access	Affordable and scalable technologies	Advanced bioengineered dressings [153]
Potential allergic reactions	Some materials or incorporated drugs may trigger hypersensitivity reactions	Skin irritation and discomfort	Hypoallergenic formulations	Adhesive and medicated patches [154]
Difficulty in large-scale commercialization	Scaling laboratory-developed technologies to industrial production is challenging	Reduced industrial implementation	Industry-academia collaboration	Experimental wound healing systems [155]
Patient compliance issues	Frequent replacement or discomfort may affect adherence to treatment	Interrupted healing process	User-friendly and flexible patch design	Thick or rigid wound dressings [156]
Environmental concerns	Disposal of synthetic and nanoparticle-containing dressings may contribute to environmental pollution	Environmental and ecological risks	Development of eco-friendly biomaterials	Synthetic polymer dressings [157]
Stability of bioactive compounds	Growth factors, peptides, and herbal extracts may lose activity over time	Reduced therapeutic efficiency	Encapsulation and stabilization techniques	Herbal and protein-loaded patches [158]
Limited clinical evidence	Many advanced wound patches lack sufficient long-term clinical trial data	Reduced clinician confidence	More multicenter clinical studies	Newly developed advanced dressings [159]
Challenges in personalized treatment	Designing patient-specific wound dressings requires advanced diagnostics and customization technologies	Difficulty in individualized therapy	Integration of AI and precision medicine	Personalized smart wound patches [160]

Regulatory and Approval Barriers

Advanced wound healing patches, particularly those containing electronics, sensors, or biologics, frequently fall under complex regulatory frameworks. These products may be classified as combination devices (drug-device-biologic), necessitating rigorous testing for safety, efficacy, and quality. Regulatory pathways differ among regions, causing delays in approval and market access. Additionally, the absence of established procedures for evaluating smart and theranostic wound systems complicates the process. To facilitate commercialization, the adoption of standardized manufacturing processes, scalable fabrication techniques, and early engagement with regulatory authorities can streamline product development and regulatory approval. Furthermore, cost-effective fabrication strategies, such as scalable biopolymer processing, automation, and additive manufacturing, may help reduce production costs and improve accessibility of advanced wound patches [161].

Stability and Storage Limitations

Bioactive wound patches containing growth hormones, stem cells, or biologics are frequently sensitive to environmental elements like temperature, humidity, and light. Maintaining stability throughout storage and transportation is a major challenge, especially in resource-constrained environments. Maintaining the functionality of embedded sensors and electronic components over time is a technical challenge [162,163].

Power Supply and Device Integration Challenges

Electronic and electroactive wound patches necessitate consistent power sources for ongoing functioning. Conventional batteries increase device bulk and limit flexibility, whereas developing self-powered solutions (such as triboelectric generators) are still in development.

Integrating power systems with flexible, biocompatible materials while maintaining patient comfort and device performance is a big engineering problem [164].

Data Management and Privacy Issues

Smart wound patches that capture and send patient data raise data security, privacy, and ethical concerns. Wireless communication systems must provide secure data delivery to prevent unauthorized access. To manage and integrate huge amounts of real-time data into clinical procedures, a strong digital infrastructure and defined protocols are necessary [165].

Significant research gaps

Advancements in wound healing patches have made significant progress, but clinical translation and commercialization remain limited due to important research gaps. Addressing these limitations is critical for converting experimental technology into safe, effective, and widely available therapeutic options for chronic and acute wound management. Lack of long-term clinical investigations on improved wound healing systems' safety, efficacy, and durability is a significant restriction. Only a few technologies have progressed to extensive human clinical trials, despite the fact that several in vitro and animal studies have produced encouraging results. It is impossible to evaluate long-term therapeutic efficacy, biocompatibility, and potential adverse effects because most currently published research uses short-term observations with small patient populations. Extensive longitudinal studies are necessary to evaluate sustained clinical performance and patient outcomes because chronic wounds, like diabetic foot ulcers and pressure sores, sometimes require protracted treatment durations [166,167]. Standardized testing methodologies and regulatory frameworks are lacking for advanced wound patches, posing substantial challenges. Different studies frequently employ different approaches to assess factors such as antibacterial activity, mechanical strength, biodegradability, drug release kinetics, and wound healing efficiency. This lack of uniformity makes it difficult to compare different technologies and causes regulatory approval to be delayed. Furthermore, multifunctional wound patches that incorporate biosensors, medication delivery systems, electronics, and nanomaterials do not easily comply with current medical device laws. Regulatory bodies thus confront difficulties in setting safety standards, quality control criteria, and approval paths for such hybrid systems. Limited understanding of nanoparticles' long-term impacts is a significant problem. Nanoparticles including silver, zinc oxide, graphene, and carbon nanotubes have strong antibacterial activity and improved therapeutic effectiveness; nevertheless, their long-term interaction with human tissues is not well understood. Potential hazards such as cytotoxicity, oxidative stress, inflammation, and nanoparticle accumulation within organs need to be investigated further. Long-term biosafety studies are required to guarantee that nanomaterial-based wound patches may be used safely in clinical practice while avoiding unforeseen systemic effects [168,169]. Completely integrated and multifunctional wound healing systems pose extra technical obstacles. Modern smart wound patches strive to integrate sensing, monitoring, drug delivery, wireless communication, and therapeutic response into a single, small platform. However, combining these disparate components while retaining flexibility, biocompatibility, stability, and user comfort remains extremely difficult. Power management, sensor reliability, material compatibility, and downsizing are important engineering challenges that must be overcome in order to create effective autonomous wound care systems [170,171]. Cost-effective and scalable manufacturing technologies are critical for global accessibility and commercialization. Many advanced wound healing technologies rely on high-cost biomaterials, nanofabrication methods, sophisticated electronics, and complex biofabrication procedures, which drive up production prices. These expenses can restrict their accessibility, particularly in low-resource healthcare environments where the prevalence of chronic wounds is typically higher. Thus, to guarantee extensive clinical use and equitable access to healthcare, scalable manufacturing methods, inexpensive biomaterials, and efficient fabrication processes are needed. Multidisciplinary collaboration among biomaterial scientists, doctors, engineers, regulatory agencies, and industry partners is needed to solve research gaps and create safe and effective wound healing technologies [172,173].

## Conclusions

In conclusion, by combining biomaterials, nanotechnology, biosensing, and smart treatment platforms into a single multifunctional strategy, next-generation wound healing patches provide a substantial improvement over traditional wound care systems. Through regulated medication distribution, infection prevention, moisture control, and real-time wound monitoring, these cutting-edge wound dressings actively contribute to the healing process in addition to offering physical protection. The possibility for individualized and adaptable wound care has been further increased by the development of stimuli-responsive and theranostic wound patches, especially in chronic and challenging-to-heal wounds such as diabetic ulcers, burns, and pressure injuries. Widespread clinical translation is still hampered by issues with manufacturing costs, extensive clinical validation, regulatory licensing, and long-term biocompatibility, despite impressive advancements. However, ongoing developments in wearable technology, 3D bioprinting, regenerative medicine, and artificial intelligence are anticipated to transform wound care approaches in the future. All things considered, next-generation wound healing patches have great potential to enhance patient outcomes, lessen healthcare costs, and open the door to completely precision-based wound care systems.
